# Building Consensus on Domains of Wellness Using Finnish and International Expert Panels: A Delphi-Method Study

**DOI:** 10.1177/08901171231204147

**Published:** 2023-09-28

**Authors:** Krista Kauppi, Eira Roos, Patrik Borg, Paulus Torkki

**Affiliations:** 1 Department of Public Health, 3835 University of Helsinki, Helsinki, Finland; 2 Aisti Health Ltd., Helsinki, Finland; 3 Medical Center Aava, Helsinki, Finland

**Keywords:** wellness, wellbeing, Delphi method, consensus study

## Abstract

**Purpose:**

The paper investigates whether we can build consensus on wellness domains and create a more universal conceptual framework for wellness.

**Design:**

A modified ranking type of Delphi method.

**Participants:**

Two separate panels consisting of 23 Finnish and 11 international experts.

**Methods:**

Panels were asked to rate the importance of 61 systematic review-based wellness domains and to eventually form a wellness model in both panels. The similarities between the resulting models were investigated and a new conceptual framework for wellness was created.

**Results:**

The Finnish model included 8 themes and 20 domains, and the international model 5 themes and eleven domains. Eight of the eleven domains were an exact match for the Finnish model (namely mental health, cognitive health, exercise, nutrition, community, life satisfaction, meaningfulness, work-life balance). There were also 2 similar domains that could be found in both models (namely self-care and lifestyle habits, social networks). A new conceptual framework for wellness was created based on these ten domains.

**Conclusion:**

The lack of consensus on the wellness construct has made it difficult to find comparable measures that could assess and improve the level of wellness of individuals, organizations, and society. This study offers a conceptual framework that can be further validated and turned into a more universal measurement instrument.

## Purpose

Increasing the level of wellness is inherently positive for individuals but also offers multiple benefits to organizations and society. These include increased labor productivity,^1,2^ reduced absenteeism,^3,4^ presenteeism, and employee turnover,^4^ reduced hospital utilization,^5^ and lower health care costs.^4,5^ Hence, it is no wonder that companies invest heavily in wellness programs^6^ while nations attempt to find comparable ways to measure wellness and use it to examine social progress.^7-9^

Despite the importance of wellness, the concept holds many definitions and there is no widely accepted agreement on what are the domains of wellness that should be measured. Wellness has been viewed as “a state of complete physical, mental, and social well-being and not merely the absence of disease or infirmity”,^10^ “an integrated method of functioning which is oriented toward maximizing the potential of which an individual is capable”,^11^ and “a way of life oriented toward optimal health and well-being in which the body, mind, and spirit are integrated by the individual to live more fully within the human and natural community”.^12^ It has been also common to use the term interchangeably with wellbeing, and confuse it with concepts such as quality of life and subjective wellbeing.^13-15^ To go around the difficulties of defining the term unanimously, it has been common to define different characteristics of wellness^11,16-18^ or to enumerate the different domains it includes.^13,19-22^ Here some of the researchers have focused on psychological factors^14,23^ while some have also included physical and social factors.^24-27^

However, different interpretations have led to a vast number of varying conceptualizations of wellness^28-32^ and little agreement on the different domains.^33^ Even though the models portray the same phenomenon – wellness – their focus might be very different. Some of the models have largely focused on psychological factors,^14,23,34^ while others have also attached importance to physical, social, and occupational factors.^21,35,36^ Hence, it is no surprise that when the models have been turned into measurement instruments, they have measured different areas of life.^12,20,24,37-39^ What makes the measurement even more challenging is the need to also account for gender, age, and cultural differences in wellness.^12,40^ With little consensus on what are the domains of wellness, it is more difficult to find and validate measures that could be used to measure and improve the level of wellness of individuals, organizations, and society.

This study applies a modified Delphi method to 1) build consensus on the domains of wellness by creating a wellness model in 2 expert panels, and 2) investigate whether we can find some common wellness domains and create a more universal conceptual framework for wellness. As this study uses 2 very different kinds of high-profile expert panels to investigate how wellness is perceived, finding similar domains not only gives health promotion researchers valuable information on the construct of wellness, but also enables the creation of a measurement instrument that could be used to investigate the level of wellness of individuals, organizations, and society.

## Methods

### Design

#### Systematic Review

Before the survey, a systematic literature review was conducted to identify different domains of wellness.^33^ The review focused on models that included at least social, physical, and psychological aspects as those are included in WHO’s definition of health, which is the most commonly cited definition of wellness.^10^ The review identified 379 unique domains from 44 wellness models and clustered them into 70 groups based on similarity. To investigate the importance of different domains of wellness, groups such as “other physical” and “miscellaneous” were excluded, resulting in 61 domain groups (shown in Supplement 1).

#### Delphi-Method

A modified ranking type of Delphi method was chosen with an a priori cut of criteria for each round and a definition of consensus.^41,42^ The number of rounds was set at 5 or 6 and a final evaluation, depending on the results of the second round. The process remained anonymous and identical for both panels and was conducted using a commercial online questionnaire tool (Elomake, Eduix Ltd). After each round, the experts received an anonymized group report, showing the results of the round as well as possible anonymized comments from other experts.

### Participants

Two separate expert panels were formed to model wellness: 1) a multidisciplinary team of Finnish professionals, and 2) an international panel of researchers. In both panels, the identities of other experts were not disclosed. Panelists were contacted via email and received an information sheet and privacy policy before participating. Consent from, and background information on the panelists were collected in the first round. The backgrounds of the experts are described in Table 1.

1) Finnish panel. The goal was to obtain as wide a view as possible of wellness. Hence, the authors mapped different disciplines that would have experience in educating or supporting people vis-à-vis their wellness. After multiple discussions, 3 high-level groups were formed with the goal of recruiting a total of 24 professionals to get a balanced and comprehensive panel. The first group was set to consist of 4 certified personal trainers and 4 work ability coaches. The second group was focused on professionals with a background in medicine. This group was set to include 4 psychologists, 2 occupational health doctors, 2 internists, 2 occupational health nurses, and 2 health center nurses. The last group included 4 human resource professionals from 4 different companies. Additional goals were to have 1) 25% of experts with a research background of some sort, 2) gender distribution close to equal, and 3) professionals from different companies and health care organizations, preferably distributed nationally. All the experts were searched from public sources i.e. known influencers of the field, medical centre websites, and company websites. The final expert panel comprised 23 professionals, minus 1 internist from the original goal of 24.
2) International panel. The systematic review^33^ revealed that there is little consensus on the domains of wellness. Hence, we decided to form another panel that would invite the international researchers who had investigated wellness or created 1 of the wellness models identified in the systematic review, to see if despite different views on wellness it would be possible to attain some degree of consensus. This panel would also represent the more scientific view of wellness. The minimum number of professionals was set at 10 as even though there is no agreement on the panel size for Delphi, panels with fewer than 10 participants are rarely conducted.^43^ Eventually, 11 researchers accepted the invitation to participate in the panel. Their backgrounds ranged from psychology, and counseling of individuals and organizations to economics and public health, with the majority being professors at different academic institutions.

**Table 1.** Characteristics of the Expert Panels.

Finnish panel		International panel	
**Gender**		**Gender**	
Male	9	Male	5
Female	14	Female	6
**Province**		**Country**	
Southern Finland	18	USA	6
Western Finland	3	UK	2
Eastern Finland	0	Australia	1
Northern Finland	2	Slovenia	1
		Finland	1
**Most descriptive background**		**Background**	
Personal trainer or physiotherapist	4	Professor	4
Work ability coach	4	Associate professor	2
Psychologist	4	Research fellow	2
Medical doctor	3	Other	3
Nurse	4		
HR manager	4		
Number of people with research background	5	Number of scientific publications	70 (111)
Professional experience gained in health care or wellness fields in years (self-rated)	21 (11)	Research experience in years (self-rated)	17 (9)
Self-rated estimate of familiarity with wellness field (average on a scale from 1 to 10)	9 (1)	Self-rated estimate of familiarity with wellness field (average on a scale from 1 to 10)	8 (2)

### Methods

#### Round 1

In the first round, the participants were asked to evaluate the importance of the 61 literature-based wellness domains using a 7-point Likert scale (1: Not important at all – 4: Neutral – 7: Extremely important). The cut-off criteria for the subsequent round were such that if 75% of the experts rated the domain as at least somewhat important (rates 5, 6, or 7), the domain would continue to the next round. To examine whether there were statistically significant differences between the panels' ratings of the importance of different areas of wellness, the Mann-Whitney U test was performed using SPSS (version 28.0.0.0).

#### Round 2

In the second round, the experts had to divide a set number of points among the remaining domains, with more points indicating higher importance. The number of points available varied depending on how many domains continued to the second round. If a maximum of 20 domains continued, the number of available points was 100; if a maximum of 40 continued, the number of points was 200; and if a maximum of 60 continued, the number of points was 300. The total number of available points was divided equally by the resulting number of domains and an average number of points was calculated. If a domain received more points than the average from the professionals, the domain continued to the next round.

#### Round 3

As a priori we were unable to know how many domains would be left for the third round, a criterion was set. If there were still over 75% (45) of the original domains left, round 2 would be repeated. However, if there were less than 45 domains left at this point, the experts were instructed to freely group and name the remaining domains based on their expertise. No domains were cut off in this round.

#### Round 4

Thematic analysis was used to identify themes from the names that the experts gave to the groups they formed. Theme refers to a higher-level description of the domains in the same group. Themes that were mentioned by more than 3 experts continued to the fourth round, where experts were asked to place the remaining domains under the most appropriate themes. Experts also had a chance to choose “None of these” if they considered that there was no suitable theme for a particular domain. If they chose that option, they were asked to name a theme that would be appropriate for that domain. The experts were also asked to evaluate on a scale from 1 to 10 how successful the naming of different groups had been. The aim was to understand whether the name described the group well enough. If the expert chose 5 or fewer, they were asked to provide an alternative name for that group. Themes that gained an average of 7.5 or higher continued to the next round and others were modified based on the experts' comments. The consensus criteria were set as follows: If at least 75% of experts placed the domain under the same theme, it was interpreted as a high consensus. If 60-75% of experts placed the domain under the same theme, it was considered to be a low consensus. If less than 60% of experts placed the domain under the same theme, it was interpreted as no consensus.

#### Round 5

The fifth round focused solely on domains that had low or no consensus in the previous round. Experts were shown the domains that received less than 75% of consensus and how the domains were distributed between different themes in the previous round. The experts were asked to choose which of the themes suited the domain better. Based on the results of round 4, some theme names could be modified.

#### Final Evaluation

Lastly, the experts were shown the end result of the Delphi process: the themes that had been formed and the domains under each theme. The experts were then asked to evaluate how satisfied they were with the formed model of wellness on a 7-point Likert scale (1: “Extremely unsatisfied” – 4: “Neutral” – 7: “Extremely satisfied”).

## Results

**Figure 1.**
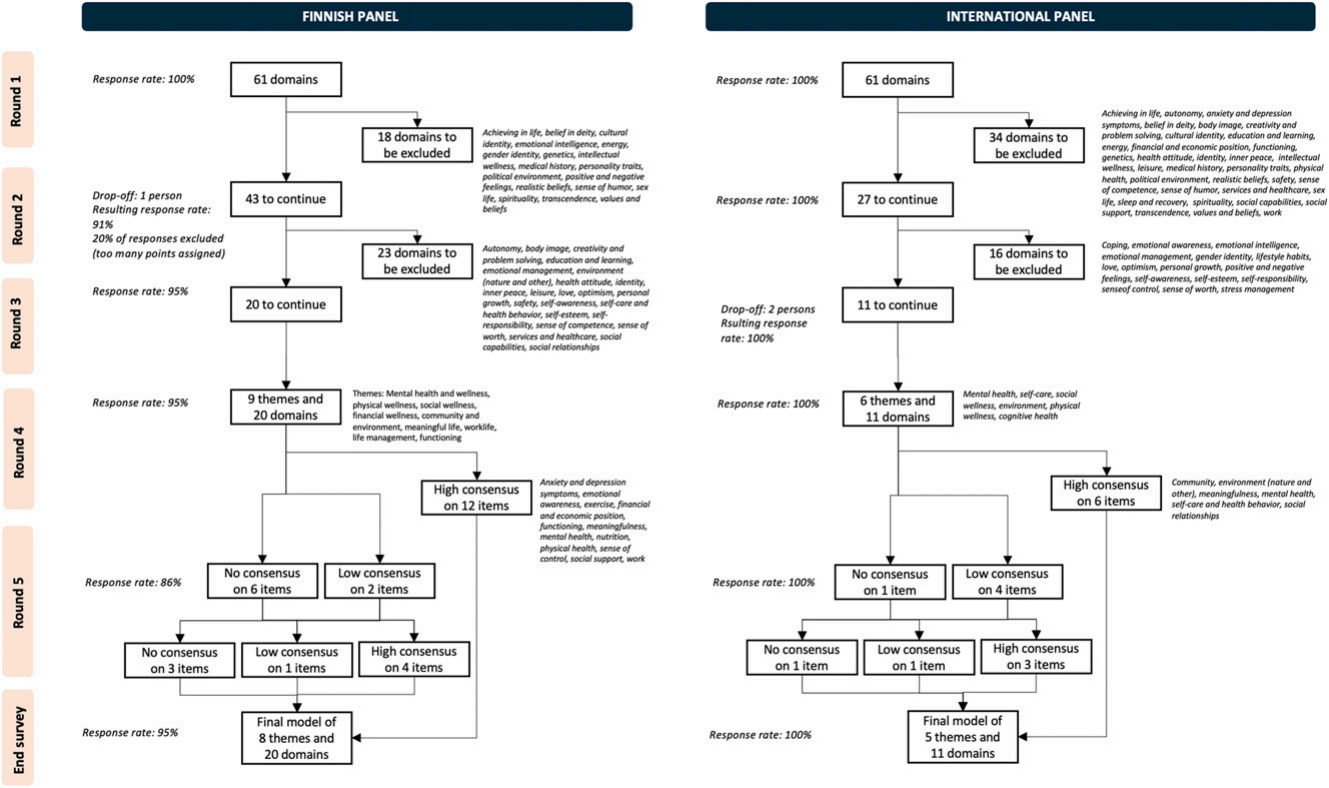
Description of the Delphi process, results of the rounds, and response rates.

The results of the modified Delphi process are described in Figure 1.

### Building Consensus on the Domains of Wellness by Creating Wellness Models in Expert Panels

#### Finnish Panel

The final wellness model consisted of 8 themes and 20 domains, where consensus on the theme under which each item belongs was reached for 80% of the items. The expert panel was satisfied with the resulting model as the average score was 5.8 and the mode 6.

#### International Panel

**Figure 2.**
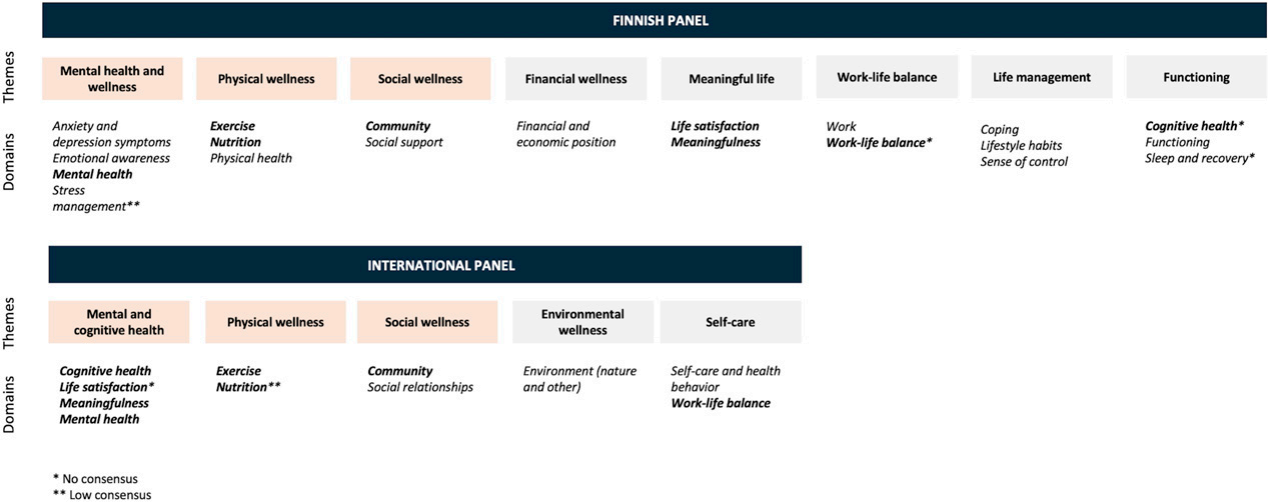
Wellness models created by the expert panels. Themes that can be found in both models are marked in different colors and domains found in both models are in boldface.

The wellness model consisted of 5 themes and 11 domains, where consensus was reached on 82% of the items. The panel was somewhat satisfied with the model with an average score of 5.1 and a mode of 6. Figure 2 presents the models created by both panels.

### Can We Find Some Common Wellness Domains and Create a More Universal Conceptual Framework for Wellness?

The model created by the international panel includes 11 different wellness domains, 73% of which found an exact match in the Finnish model. Furthermore, the international panel included “Self-care and health behavior”, which is somewhat similar to the Finnish panel's “Lifestyle habits”. It also included “Social relationships”, which links to the Finnish panel's “Social support”. If these 2 are also taken into consideration, we can conclude that 10 out of 11 domains in the international panel’s model can be found in the Finnish model.

**Figure 3.**
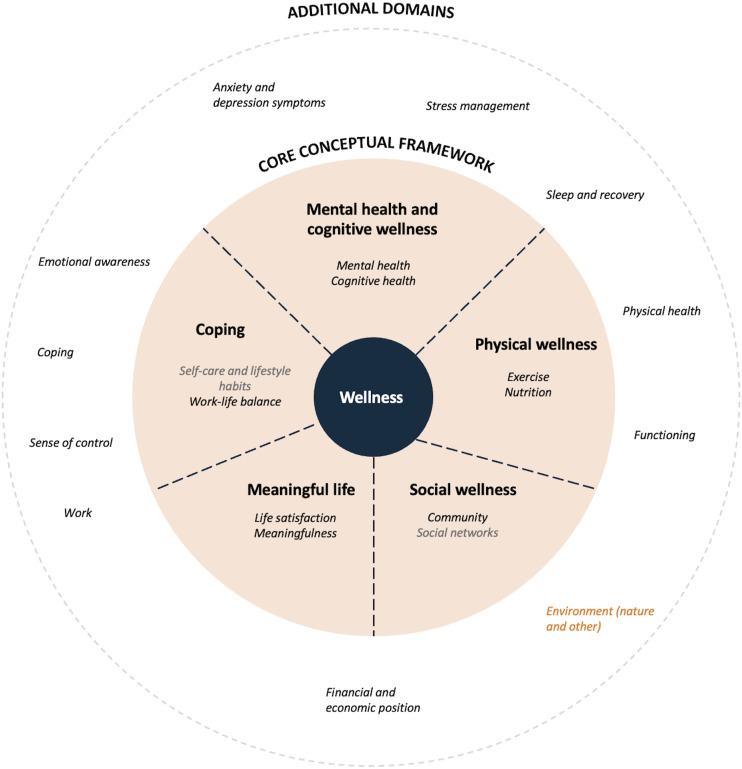
Combined conceptual framework of wellness based on the similarities identified by the expert panels. The inner circle depicts the core framework, which consists of the 8 identical domains (marked in black) and the 2 similar domains (marked in gray) identified by both panels. The outer circle portrays the domains that were identified by either of the 2 panels, with domains identified by the international panel marked in orange.

There were also 3 similar themes in both panels, namely “Mental health”, “Physical wellness”, and “Social wellness”. Both panels had placed “Mental health” under the “Mental health” category, “Exercise” and “Nutrition” under “Physical wellness”, and “Community” under “Social wellness”. Based on this, a suggestion for a more universal conceptual framework for wellness is presented in Figure 3.

As there were multiple similarities between the models, additional statistical testing of the first-round results was performed to see if the opinions were somewhat in line since the start. When looking at the results of the first round, where the experts on both panels were instructed to rate the importance of each domain of wellness, no statistically significant differences were found between the panels (Supplement 1).

## Discussion

We aimed to build consensus on the construct of wellness by creating 2 separate wellness models in 2 very different multidisciplinary expert panels and by investigating whether similar domains appear. We decided to use the Delphi method as it has been deemed suitable for situations where the issue is highly complex and knowledge is incomplete.^41,44,45^ We found that most of the domains and themes in the international model could also be found in the broader Finnish wellness model. Hence, this paper provides an interesting perspective on how wellness is modeled and offers ten domains of wellness that could be used to create a measurement instrument for further validation.

### Building Consensus on the Domains of Wellness by Creating Wellness Models in Expert Panels

The Finnish model included 8 themes and 20 domains, while the model by the international panel included 5 themes and eleven domains. The former is more than in the previous literature, where the number of domains has ranged from 1 to 15^29,30^ with an average of 5 domains, and a median of 8.^33^ This makes both models relatively large compared to the existing literature.

We can observe some interesting differences between the models. For instance, the Finnish panel produced more domains related to mental and physical health, life management, and functioning. These included anxiety and depression symptoms, emotional awareness, stress management, physical health, coping, sense of control, functioning, and sleep and recovery. Furthermore, the Finnish panel attached importance to one’s financial and economic position, and work, while the international panel highlighted the importance of the environment. At this point, we can only speculate about the reason for these differences and state based on our own expertise that the domains suggested by the Finnish panel seem to reflect the latest trends and topics around employee wellness. More research would be required to ascertain whether these differences really stem from the geographical and cultural differences of the panel or a possible scientist-practitioner gap,^46^ as the Finnish panel consisted mainly of practitioners, while the international panel included high-profile researchers.

Furthermore, the panelists also provided some excellent conceptual comments to ponder concerning the models. For instance, some panelists wondered whether anxiety and depression symptoms were an appropriate name for the domain and whether life satisfaction can be placed under any specific theme as it is a composite measure. These issues could warrant further debate. Meanwhile, it is good to note that a high degree of consensus on the placement of different domains was reached in both panels in over 80% of cases. The level set for high consensus conforms with the median threshold found in other studies.^47,48^ Considering the consensus rates and mode of 6 for the satisfaction score (7-point Likert scale = “Satisfied”), we can safely conclude that the created models represent the panelists' opinions and expertise.

### Can We Find Some Common Wellness Domains and Create a More Universal Conceptual Framework for Wellness?

There have been a vast number of different conceptualizations of wellness, with little consensus on the domains. A recent systematic review examined how different wellness models were created and noticed that only one-third of the models were based on previous literature.^33^ This study attempted to take the first steps towards identifying some common themes and testing the methodology by using 2 different panels to rank 61 domains of wellness in 2 independent anonymous modified Delphi processes. We were able to identify 8 identical domains, 2 similar domains, and 3 similar themes. This means that 73% of the domains in the smaller model (international panel) could be found in the larger Finnish model, and if the 2 similar domains were also accounted for, the percentage would rise to 91%. Based on this, we could agree on ten domains of wellness and form a conceptual framework for wellness for further validation (core conceptual framework in Figure 3). The framework could be further improved by placing the additional domains under the themes presented in the core. However, this review focuses only on the core domains, placing emphasis on the similarity of the models.

Considering the current lack of consensus on the domains of wellness, this similarity between the models is somewhat surprising, particularly as it does not seem to be based on the occurrence rate of domains in previous models. A previous systematic review investigated the occurrence of different domains in 44 different models and, for instance, “cognitive health” was mentioned only 4 times compared to “positive and negative feelings” being mentioned 22 times.^33^ However, as expected, we found 3 similar themes in both models. These were “Mental health”, “Social wellness”, and “Physical wellness”, which all stem from the most cited definition of health.^10^

We can see similarities between our core framework and multiple existing frameworks that are used to investigate wellness and social progress. Our framework resembles WHO’s definition of health by including social, physical, and psychological domains, but also includes coping, namely the “ability to adapt and self-manage”, from the revised version of the definition of health.^10,49^ It also has similarities with the UK’s measure of personal wellbeing, the European Union’s quality of life model, and the OECD well-being framework.^8,9,50^ These similarities are vital as the basis of a unified measure is agreement on the areas of measurement. However, as there are still differences, more work is needed to get closer to operationalization. It would be worthwhile investigating whether our core framework could work as the basis for a more unified measure of wellness that could be expanded with additional important domains from existing international frameworks. We acknowledge the difficulty but stress the importance of this kind of harmonization work and point to the fact that harmonization has been achieved in complex measures such as the quality-adjusted life year measure, which is used to assess health status in different conditions.

Certain factors should be considered when using the new conceptual framework for wellness. As 2 of the domains were not an exact match in both models, we decided to suggest a combined name for “social relationships” and “social support”, as well as “lifestyle habits” and “self-care and health behavior”. We acknowledge that our suggested terms “social networks” and “self-care and lifestyle habits” might not completely capture the original domains, and therefore encourage fellow researchers to investigate more appropriate names for these entities. We also recommend summarizing the vast existing literature and setting clear definitions for each theme and domain. The next step towards operationalization would be to gather all the different measurement methods systematically for each of the domains, evaluate their usefulness, and build a first version of the instrument for further validation. For instance, there are many validated instruments for mental health.^51,52^

Furthermore, it would be beneficial to experiment with a different kind of Delphi process to see whether the results are reproducible, such as using multiple different groups in the same Delphi process.^53^ We also recommend combining the national and international panel in the future when building models. Although for the purposes of this review, it was justified to keep them separate as there was a need for a national model and cultural differences in wellness have been identified.^12^ If the results were reproducible, the conceptual framework could be tested in larger-scale studies to observe how the perspective on wellness varies between age groups, genders, and cultures, for instance. We also acknowledge the time dependency of the model. Our perception of wellness evolves, meaning that there might be some new domains in the future, while some existing ones might become less relevant.

### Strengths and Limitations of the Study

We identified some limitations that might have affected the results of this review. Despite the large systematic review, some experts felt that certain domains were missing. Hence, it might be beneficial to modify the process and give experts a chance to suggest new domains as well. Moreover, the process of ranking could be enhanced, especially in the second round, which was considered to be difficult and was prone to error as the tool that was used allowed 1 to allocate more than a set number of points. Nevertheless, this error occurred in only 13% of cases and in only 1 round of the Finnish panel, so we do not consider that this had a significant effect on the results. As there were also some additional struggles in using the tool assigned by the University, resulting in 1 expert dropping out of the international panel, we would recommend using a different tool in the future. However, despite the process being complex and time-consuming, we experienced relatively low drop-out rates (4% and 18%). Furthermore, the number of participants limited our capabilities to do statistical testing.

Furthermore, some experts were hoping for some workshops or meetings where they could discuss the domains and model more profoundly. This could have been beneficial, especially when naming the themes. Nonetheless, this should be carefully considered and balanced as removing anonymity in panels with high-profile influencers such as in this case can also lead to bias.^54,55^ There was a clear need to have 2 separate panels in this research, but the framework could be improved by using a combined panel of experts. Lastly, it would have been beneficial to have clear descriptions for each domain, but as these did not exist, they would have had to be created, which could have caused bias in the end result. Nonetheless, the strengths of this study lie in it being based on a wide systematic review that identified the domains of wellness, a very strict a priori defined Delphi process with clearly predefined consensus rates, and 2 different expert panels.

### Implications for Health Promotion Practice and Research

This study provides health promotion researchers with a perspective on how wellness is modeled and offers a new conceptual framework for further validation, consisting of 8 identical domains and 2 similar domains deriving from 2 distinct expert panels. As this review focused only on experts' views on wellness, it would be important to also investigate how laypeople view the importance of different areas of wellness compared to experts. Furthermore, the conceptual framework should be turned into a measurement instrument, which would enable investigation of the differences between countries, cultures, age groups, and genders, as well as how different domains interrelate with each other. With a measurement instrument, we could also investigate how the different areas of wellness are linked to high burdens of individuals, organizations, and societies such as burnout, generalized anxiety disorder, and depression. We hope that this study encourages health promotion researchers to continue research on the complex nature of wellness and to find systematic ways to develop wellness models to be taken into practice.

## Conclusion

This study investigated the construct of wellness by using 2 separate multidisciplinary expert panels to form wellness models in a modified Delphi process. In both models, the following domains were found: “Mental health”, “Cognitive health”, “Exercise”, “Nutrition”, “Community”, “Life satisfaction”, “Meaningfulness”, and “Work-life balance”. Furthermore, we found 2 similar domains in both models, which were named “Social networks” and “Self-care and lifestyle habits”. We argue that it is important to reach agreement on domains of wellness in order to create a more universal measurement instrument. Considering that we still use many different frameworks to measure national wellbeing and social progress, we have taken a step toward harmonization by offering ten domains that can be used as the basis for a more unified measure of wellness. We encourage other researchers to validate and expand on our core framework, and to investigate whether it could be used to measure and improve the level of wellness of individuals, organizations, and society at large.

**SO WHAT?**

**What is already known on this topic?**

There is a clear need to measure individuals' wellness to examine social progress. Many countries have started to incorporate wellness measures into their measurement instruments but often end up measuring different aspects of wellness.

**What does this article add?**

This paper is 1 of the scarce attempts to build consensus on the construct of wellness. It uses Delphi method in 2 distinct expert panels. The result is ten domains of wellness that could be used to create a measurement instrument for further validation.

**What are the implications for health promotion practice or research?**

Higher levels of wellness have been shown to have multiple positive benefits to individuals, organizations, and society. To see how political decisions or environmental, technological, and global changes affect our population's wellness, we need to find comparable measures of wellness. This paper offers ten domains of wellness that can be used as a basis for a more unified measure of wellness.

## Supplemental Material

Supplemental Material - Building Consensus on Domains of Wellness Using Finnish and International Expert Panels: A Delphi-Method Study Click here for additional data file. Supplemental Material for Building Consensus on Domains of Wellness Using Finnish and International Expert Panels: A Delphi-Method Study by Krista Kauppi, Eira Roos, Patrik Borg, and Paulus Torkki in American Journal of Health Promotion
