# Supplementary material for: Building Consensus on Domains of Wellness Using Finnish and International Expert Panels: A Delphi-Method Study
Source: Am J Health Promot. 2023 Sep 28;38(2):228–37. doi: 10.1177/08901171231204147 (PMC10802088; doi:10.1177/08901171231204147)
Supplement: Supplemental Material - Building Consensus on Domains of Wellness Using Finnish and International Expert Panels: A Delphi-Method Study [file sj-pdf-1-ahp-10.1177_08901171231204147.pdf]

Supplement 1 – Results of the Mann-Whitney U test comparing differences between the Finnish and international panels

Domain	p-value	Domain	p-value
Achieving in life	0.258	Mental health	0.424
Autonomy	0.537	Nutrition	0.828
Anxiety and depression symptoms	0.490	Optimism	0.800
Belief in deity	0.800	Personal growth	0.176
Body image	0.772	Personality traits	0.856
Cognitive health	0.291	Physical health	0.424
Community	0.942	Political environment	0.586
Coping	0.091	Positive and negative feelings	0.114
Creativity and problem solving	0.114	Realistic beliefs	0.326
Cultural identity	0.424	Safety	0.490
Education and learning	0.490	Self-awareness	0.143
Emotional awareness	0.828	Self-care and health behavior	0.308
Emotional intelligence	0.744	Self-esteem	0.561
Emotional management	0.291	Self-responsibility	0.913
Energy	0.856	Sense of competence	0.344
Environment, nature and other	0.176	Sense of control	0.363
Exercise	0.468	Sense of humor	0.744
Financial and economic position	0.586	Sense of worth	0.403
Functioning	0.133	Services and healthcare	0.885
Gender identity	0.308	Sex life	0.363
Genetics	0.326	Sleep and recovery	0.243
Health attitude	0.274	Spirituality	0.363
Identity	0.153	Social capabilities	1.000
Inner peace	0.612	Social relationships	0.383
Intellectual wellness	0.091	Social support	0.326
Leisure	0.243	Stress management	0.586
Life satisfaction	0.445	Transcendence	0.291
Lifestyle habits	0.717	Values and beliefs	0.913
Love	0.663	Work	0.744
Meaningfulness	0.717	Work-life balance	0.513
Medical history	0.856		
